# A modulatory role of ASICs on GABAergic synapses in rat hippocampal cell cultures

**DOI:** 10.1186/s13041-016-0269-4

**Published:** 2016-10-19

**Authors:** Maksim Storozhuk, Elena Kondratskaya, Lyudmila Nikolaenko, Oleg Krishtal

**Affiliations:** 1 Bogomoletz Institute of Physiology, Bogomoletz st. 4, Kiev, Ukraine; 2 State Key Laboratory of Molecular and Cellular Biology, Bogomoletz st. 4, Kiev, Ukraine

**Keywords:** Protons, Synaptic transmission, Amiloride, Diminazene, GABA

## Abstract

Rapid acidification occurring during synaptic vesicle release can activate acid-sensing ion channels (ASICs) both on pre- and postsynaptic neurons. In the latter case, a fraction of postsynaptic current would be mediated by cation-selective acid-sensing ion channels. Additionally, in both cases, activation of acid-sensing ion channels could modulate synaptic strength by affecting transmitter release and/or sensitivity of postsynaptic receptors. To address potential involvement of acid-sensing ion channels in mediation/modulation of synaptic transmission at hippocampal GABAergic synapses, we studied effects of three structurally different blockers of acid-sensing ion channels on evoked postsynaptic currents using the patch-clamp technique. We found that GABAergic postsynaptic currents, recorded below their reversal potential as inward currents, are suppressed by all the employed blockers of acid-sensing ion channels. These currents were suppressed by ~ 20 % in the presence of a novel blocker **5b** (1 μM) and by ~30 % in the presence of either amiloride (25 μM) or diminazene (20 μM). In the same cells the suppression of postsynaptic currents, recorded above their reversal potential as outward currents was statistically insignificant. These results imply that the effects of blockers in our experiments are at least partially postsynaptic. On the other hand, in the case of mediation of a fraction of postsynaptic current by acid-sensing ion channels, an increase of outward currents would be expected under our experimental conditions. Our analysis of a bicuculline-resistant fraction of postsynaptic currents also suggests that effects of the blockers are predominantly modulatory. In this work we present evidence for the first time that acid-sensing ion channels play a functional role at hippocampal GABAergic synapses. The suppressing effect of the blockers of acid-sensing ion channels on GABAergic transmission is due, at least partially, to a postsynaptic but (predominantly) modulatory mechanism. We hypothesize that the modulatory effect is due to functional crosstalk between ASICs and GABA_A_-receptors recently reported in isolated neurons, however, verification of this hypothesis is necessary.

**Electronic supplementary material:**

The online version of this article (doi:10.1186/s13041-016-0269-4) contains supplementary material, which is available to authorized users.

## Introduction

ASICs are abundant in many brain areas and are known to have important physiological functions. However, because of rapid desensitization of ASIC-mediated currents, synapses are among the few places where they can be activated under physiological conditions and, thus, mediate their physiological role.

While under physiological conditions the brain’s extracellular pH is reasonably constant, neural activity can induce transient and localized pH fluctuations, in particular, due to release of synaptic vesicles which have a pH of ~5.2–5.7. Indeed, there is evidence indicating that acidification occurs at synaptic cleft in several types of synapses in different brain structures [1–4]. Moreover, it has been recently shown that in the lateral amygdala, protons act as neurotransmitter by activating acid-sensing (proton-gated) channels (ASICs) and regulate synaptic plasticity in this structure [5]. Although no direct involvement of ASICs in synaptic transmission (synaptically activated ASIC mediated currents) was detected in the hippocampus, it has been well documented that ASICs are involved in regulation and plasticity of glutamatergic synaptic transmission in the hippocampus [6–8]. However, the synaptic cleft acidification occurs also at inhibitory GABAergic synapses [1], and selective deletion of ASIC1a in GABAergic cells, has important functional consequences [9]. Finally, functional crosstalk between ASICs and GABA_A_-receptors has been recently reported [10, 11]. This notwithstanding, possible involvement of ASICs in the regulation of GABAergic transmission is still poorly investigated. We have started to address this question by examining possible effects of several ASIC blockers on evoked GABAergic PSCs in hippocampal cell culture.

It should be noted that ASICs are in any case naturally present in hippocampal neurons as detail studied in [8, 12–16].

Below we briefly outline information related to our work.

1. hippocampal ASIC-like current is due to a mixture of homomeric ASIC1a channels and heteromeric channels [8, 12–16]. An estimate of *functional* (membrane-located) ASIC subtypes in different brain structures has been provided in recent elegant work [16]. According to this estimate the proportion of *functional* ASICs in acute hippocampal tissue is as following: 1a:1a:1a 33.2 %; 1a:1a:2a 44.2 %; 1a:2a:2a 19.6 %; 2a:2a:2a 2.9 % [16].
2. The density of proton-activated currents (evoked by pH shift to 5) is about 17 -20 pA/pF in hippocampal pyramidal neurons [8, 12]. The density of proton-activated currents in hippocampal inhibitory interneurons was also estimated and compared with that in pyramidal cells [17]. It was found that in basket cells the density of ASIC current (0.12 pA/μm^2^) is about the same as in pyramidal neurons (0.11 pA/μm), however it is substantially higher in oriens lacunosum-moleculare (O-LM) interneurons (0.75 pA/μm^2^) [17]. ASIC currents of these three cell types were blocked (by more than 50 %) in presence of amiloride at 10 μm concentration [17].

Given that ASICs are involved in mediation/modulation of synaptic transmission at hippocampal GABAergic synapses, we can draw the following conclusions:

1) Weaker effects of ASIC blockers specific to homomeric ASIC1a channels on PSCS should be expected. 2) As compared to amygdala neurons, weaker effects should be expected in most of the hippocampal neurons, because the density of proton-activated currents is higher in amygdala [8].

## Methods

Animals: Albino Wistar rat pups were housed under a constant 12/12 hour light/dark cycle at 22–24 °C in the institutional animal facility and removed from the litter no more than half an hour before anaestesia. All procedures used in this study were approved by the Animal Care Committee of Bogomoletz Institute of Physiology and conform to the Guidelines of the National Institutes of Health on the care and use of animals.

For studying synaptic responses we used cultures of rat hippocampal neurons, a preparation that enables the recording of the responses evoked by a single presynaptic neuron stimulation relatively easily. Cell cultures were prepared as described previously [18]. All cultures were kept at 36 °C in humidified air with 5 % CO_2_ and were used for the experiments 14-22 days after plating. Unless otherwise noted, relatively low-density areas of coverslips with cultured cells (2-5 neurons in 400-μm diameter view-field) were selected for the experiments. Synaptic responses were evoked by applying voltage pulses (0.2-1 ms, 20-100 V) to an extracellular electrode (a patch electrode filled with the extracellular solution) positioned in the vicinity of the presynaptic neuron soma or neurite. Such an approach allows local (“down to” a single synaptic bouton) extracellular stimulation [19, 20]. A standard whole-cell patch-clamp technique was applied to record responses (IPSCs) from postsynaptic neurons. In the framework of this work we focused on similarities of GABAergic synapses, regardless of the nature of postsynaptic cells (GABAergic versus glutamatergic). Nevertheless, postsynaptic neurons, used in our experiments were mainly excitatory. About 80 % of postsynaptic neurons were glutamatergic by virtue of triangular-shaped cell bodies, a typical feature of pyramidal neurons. About 10 % of postsynaptic neurons were definitely GABAergic, because brief depolarization (from Vh-70 mV) of their soma evoked autaptic GABAergic responses. The remaining ~10 % of cells were probably a mixture of both types.

**Fig. 1:**
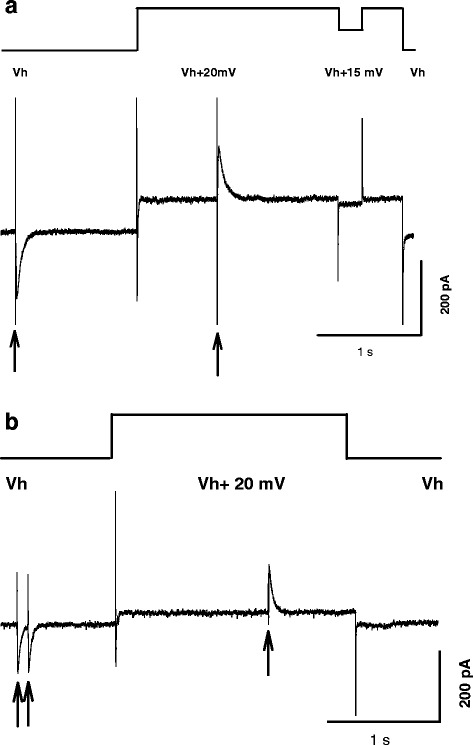
Experimental protocols used in most of the experiments. During each sweep a presynaptic neuron was stimulated twice, firstly when the membrane potential in the postsynaptic cell was clamped 10-15 mV below PSC reversal potential for a given synaptic connection (typically –45 mV), and secondly when the membrane potential was shifted by 20 mV (typically to –25 mV). Sweeps were collected every 4 seconds. Stimulations of a presynaptic neuron are marked by arrows. Voltage in the postsynaptic neuron is schematically shown in upper panels, and currents in the lower. **a** Experimental protocol used in most of the experiments with **5b. b** Experimental protocol used in the experiments with amiloride and diminazene. This protocol is similar to that shown in A, but during each sweep a pre-synaptic neuron was stimulated three times – two currents were recorded as inward and one as outward. The upper panel illustrates voltage protocol, and the lower panel currents recorded in a postsynaptic neuron

Slow (as compared to glutamatergic) evoked responses were assumed to be mediated by GABA_A_ receptors since they reversed reasonably close to the chloride equilibrium potential. The intracellular solution contained the following (in mM): Cs gluconate 100, CsCl 30, MgCl_2_ 4, Na_2_ATP 4, ethylene glycol tetraacetic acid (EGTA) 10, N[2-hydroxyethyl]piperazine-N’-[2-ethanesulfonic acid] (HEPES) 10. In most of the experiments the extracellular solution contained the following: (in mM): NaCl 140, KCl 4, CaCl_2_ 2, MgCl_2_ 1, HEPES 2, glucose 10 (‘HEPES 2 –solution’), in some series of experiments, however, higher concentrations of HEPES were used (3 or 10 mM); pH of all solutions was 7.4. Unless noted otherwise, 10 μM of 6-cyano-7-nitroquinoxaline-2,3-dione (CNQX) and 50 μM of DL-2-amino-5-phosphonovaleric acid (APV) were added to extracellular solution to block ionotropic glutamate receptors to study pharmacologically isolated GABAergic responses. Small volumes of tested antagonists were gently added directly in a corner of a static bath (2 ml) to obtain a final desired concentration. Except for a novel antagonist of ASIC1a 2-oxo-2H-chromene-3-carboxamidine derivative **5b** (developed by joint efforts of scientists from Institute of Organic Chemistry NAS and A.A. Bogomoletz Institute of Physiology NAS [21], referred to hereafter as **5b**), chemicals were obtained from Sigma-Aldrich company. **5b** was used at 1 μM concentration, which completely blocks ASIC-mediated currents evoked by mild (pH 6,7) acidification. In particular, rASIC1a-like currents in *hippocampal neurons* evoked by mild (pH 6,7) acidification were decreased by **5b** (100 nM) up to 9.39 ± 2.9 % of control values (Please, see supporting information for [21]). Experimental membrane potentials reported here were corrected for liquid junction potentials as suggested in an earlier study [22]. In most of the experiments we used experimental protocol similar to those described in Fig. 1a or Fig. 1b. During each sweep a presynaptic neuron was stimulated twice; first when the membrane potential in the postsynaptic cell was clamped 10-15 mV below IPSC reversal potential for a given synaptic connection (typically –45 mV), and second when the membrane potential was shifted by 20 mV (typically to –25 mV) (Fig. 1a). The above protocol was used in the majority of the experiments with **5b**. A slightly modified protocol was used to study the effect of amiloride and diminazene (Fig. 1b). In this protocol, during each sweep the presynaptic neuron was stimulated three times – two currents were recorded as inward and one as outward. Both protocols enabled the further estimation of the reversal potential by extrapolation. The reversal potential of the evoked synaptic currents in ‘HEPES 2 –solution’ was -33,3 ± 1,20 mV (*n* = 18), and the theoretically calculated equilibrium potential for chloride ions with intracellular and extracellular salines applied was -34,6 mV.

In most of experiments with bicuculline the membrane voltage was clamped at –55 –70 mV to increase the amplitude of PCSs.

Digitized currents were analyzed using ANDATRA software kindly provided by Yaroslav Boychuk (A.A. Bogomoletz Institute of Physiology, Kiev, Ukraine). Unless noted otherwise, the data are presented as a mean ± S.E.M; Student’s t-test was used for statistical comparisons.

## Results

Rapid acidification occurring during synaptic vesicle release can activate ASICs both on pre- and postsynaptic neurons. In the latter case, a fraction of postsynaptic current would be mediated by cation-selective ASICs, as it was previously demonstrated for the lateral amygdala neurons [5]. It should be noted however, that in both cases activation of ASICs could also modulate synaptic strength by affecting transmitter release and/or sensitivity of postsynaptic receptors. To address these possibilities we studied effects of three structurally different ASIC blockers on evoked postsynaptic currents. In particular we studied potential effects of compound **5b**, reported as a quite selective ASIC blocker [21].

### The effect of compound 5b on GABAergic PSCs is likely to be due to predominantly modulatory action related to rapid synaptic acidification

#### Effect of compound 5**b** on GABAergic PSCs in HEPES 2 solution

If a fraction of postsynaptic current at hippocampal GABAergic synapses is mediated by cation-selective ASICs, ionic composition of PSCs will be comprised by both cations and Cl^-^ anions. Given that the reversal potentials for cations and Cl^-^ are not the same (for particular solutions) the different effects of ASIC blocker may be expected on inward and outward currents. We designed our experiments to examine this possibility. In the same series of experiments we recorded PSCs at GABAergic synapses below their reversal potential as inward currents, and above the reversal potential, as outward ones. In both cases, however, membrane potential was below the reversal potential for Na^+^ (see Methods for details). Under these conditions, if a fraction of postsynaptic current is mediated by cation-selective ASICs, it would be expected that the block of ASICs will decrease the inward current, but *increase* the outward ones.

Since it has been reported previously that 10 mM of HEPES is higher than the physiologically relevant concentration of proton buffer [1, 3], the first series of experiments was done in 2 mM HEPES solution. As illustrated in Fig. 1a, the sweeps were collected every 4 seconds. After at least 50 control sweeps, **5b** was added to the recording chamber to reach the final concentration of 1 μM, then at least 80 more sweeps were collected. Since cell-to-cell IPSC amplitudes were quite variable, the amplitude values were normalized to the control value (average amplitude of 20 PSCs before the drug application) in each single experiment and then the results from different experiments were pooled. In the following graphs sequential averages of 10 PSCs are plotted versus time.

**Fig. 2:**
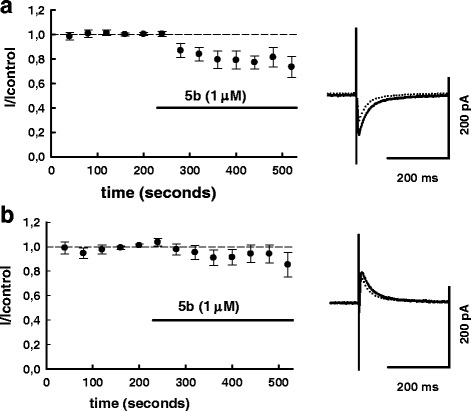
**5b** (novel blocker of ASICs) suppresses inward GABAergic currents in ‘HEPES 2’ solution. In the same series of experiments during each sweep evoked PSCs were recorded below their reversal potential as inward currents (**a**), and above the reversal potential, as outward ones (**b**) - see Methods for details. Superimposed traces of original current traces (averages of 10 sequential PSCs) before (solid lines) and after (dotted lines) **5b** (1 μM) application are shown on the right panel, summary graphs (*n* = 8) are shown on the left panel. PSC-amplitudes were normalized to control values (average of 20 PSCs preceding drug application). In control, absolute amplitudes of inward and outward currents (mean ± S.D) in this series of experiments were: -194.35 ± 93,1 pA; 214.8 ± 175.4 pA

We found that following application of **5b**, the inward currents were substantially decreased as compared to control (Fig. 2a, b). On average, the decrease in the inward current amplitude was 19.9 ± 5.9 % (*n* = 8), and the inhibitory effect was statistically significant (*P* < 0.02; T = -3.186003; df = 7; paired Student’s t-test). At the same time there were no statistically significant changes of the outward currents; on average the amplitude of outward PSCS in **5b** presence was 93.9 ± 5.7 % of control (*P* >0.3; T = -1.04; df = 7; paired Student’s t-test).

Thus, while the effect of **5b** on the inward currents could be explained by the direct involvement of ASICs in PSC generation as observed in the amygdala [5], the lack of an increase of the outward currents suggests that the effect of **5b** is predominantly modulatory. Lack of apparent *shift* of estimated PSCs reversal potential (-0.75 ± 0.26 mV, *n* = 8) after application of **5b** also supports this suggestion. (see Methods for details of estimating PSCs reversal potential). Nevertheless, to further verify this point we tried to pharmacologically isolate/enhance a fraction of synaptic current that is not mediated by GABA_A_-receptors (residual current) and study some of its properties. Bicuculline (20 μM) was used to block (most of) GABA_A_-receptors in this series of experiments. Since the IC50 value for bicuculline effect on GABA(A) receptors for is estimated as 2.7 μM [23], approximately 90 % blockade is anticipated for 20 μM concentration.

#### ASICs do not mediate a substantial fraction of residual currents at hippocampal GABAergic synapses

Generally, bicuculline (20 μM) did not completely block evoked responses, recorded in the presence of APV (50 μM) and CNQX (10 μM).

**Fig. 3:**
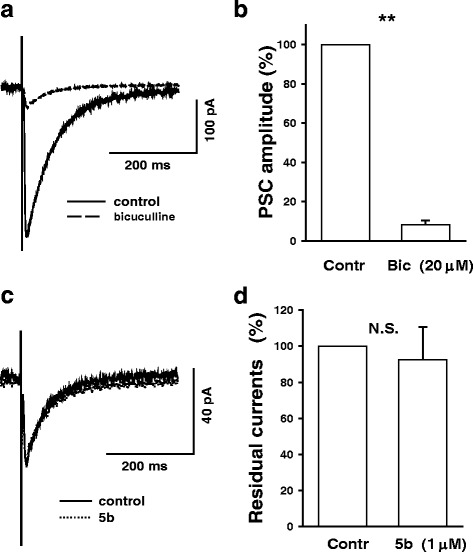
ASICs do not mediate substantial fraction of PSCs at hippocampal GABAergic synapses. Top panel: only a small fraction of evoked GABAergic PSCs is bicuculline-resistant. **a.** An example of original current traces (averages of 10 sequential PSCs) before and after bicuculline (20 μM) application. This is an example of a relatively large residual current. For the experiments described below, we selected cells with relatively large residual currents. **b**. Summary graph (*n* = 20). In control (before bicuculline), absolute amplitude of currents (mean ± S.D) was: -336.5 ± 144.7 pA. Bottom panel: **5b** does not strongly affect bicuculline-resistant (residual) currents at GABAergic connections. **c**. Superimposed traces of original current traces (averages of 10 sequential PSCs) before and after **5b** (1 μM) application. **d.** Summary graph (*n* = 5). In control (before **5b** application), absolute amplitude of currents (mean ± S.D) was: -45.8 ± 21.6 pA

For the experiments described below we selected cells with large residual currents as illustrated in Fig. 3a. Figure 3b summarizes data obtained in experiments with bicuculline. On average, amplitude of the bicuculline-resistant fraction of evoked PSCs (residual currents) was 8.23 ± 2.24 % (*n* = 20, range 0-34 %). We failed to find a systematic effect of 5 b (1 μM) on these currents - see Fig. 3c, d. On average, in the presence of 5 **b** the average amplitude of residual currents was 92.9 ± 18.1 % of control (*P* = -0,39; paired t-test *n* = 5). On the other hand, these currents were strongly affected in the presence of suramin, a non-selective P2X antagonist. We found that suramin at 200 μM concentration (Additional file 1: Figure S1) decreased residual currents to 16.9 ± 4.3 % of control (*P* < 0.01; paired t-test, *n* = 4); and to 66.5 ± 14 % (*n* = 4) at 20 μM.At the same time, it should be borne in mind that: a) suramin (500 μM) does not affect ASIC currents in hippocampal neurons [24]; b) it is unlikely that the residual currents under our experimental conditions were mediated by P2X receptors. The latter point can be supported by our previous observation that suramin (20 μM) substantially reduced the amplitude of currents evoked by *exogenous* GABA application (GABA-currents) [25]. On average, GABA-currents were reduced to 41.9 ± 2.9 % of control (*n* = 4; *P* < 0.01; paired t-test) in presence of 20 μM of suramin [25]. Additionally, in some experiments with bicuculline we used a protocol similar to that illustrated in Fig. 1a, which enabled us to obtain an estimate of the PSC reversal potential before and after bicuculline application, and thus a possible *shift* of the reversal potential evoked by bicuculline application. There were no pronounced shifts of the PSC reversal potential. On average, the shift was -1.29 ± 1.31 mV (*n* = 5). These results support the idea that under our experimental conditions, even in the presence of bicuculline, synaptic currents are still predominantly mediated by chloride ions.

#### The effect of compound **5b** on GABAergic PSCs is attenuated in HEPES 10 solution

To check whether the effect of **5b** is related to endogenously occurring acidification, in a separate series of experiments we studied the effect of this compound in extracellular solution with enhanced concentration of proton buffer (HEPES). Such an approach has been previously justified in several studies [1, 3, 26]. In particular, we studied the effect of **5b** (1 μM) in the extracellular solution containing 3 mM of HEPES, concentration close to level of physiological buffering [3]. We have found that in the extracellular solution with 3 mM of HEPES (*n* = 5), **5b** had a reduced effect on inward currents. On average, the decrease of inward current amplitude was 14.3 ± 4.9 %, but it was still statistically significant (*P* < 0.05; T = -2.9; df = 4; paired Student’s t-test, not illustrated).

**Fig. 4:**
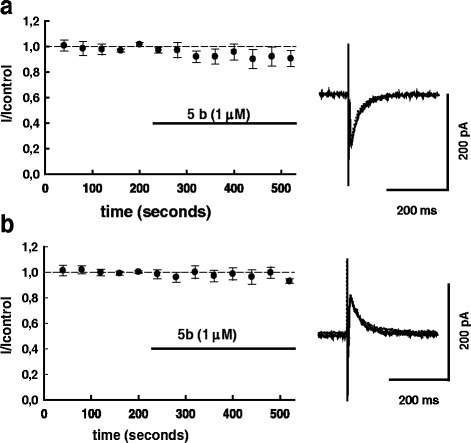
**5b** (novel blocker of ASICs) has little effect on inward GABAergic currents in ‘HEPES 10’ solution. In the same series of experiments during each sweep evoked PSCs were recorded below their reversal potential as inward currents (**a**), and above the reversal potential, as outward ones (**b**). Superimposed traces of original current traces (averages of 10 sequential PSCs) before (solid lines) and after (dotted lines) **5b** (1 μM) application are shown on the right panel, summary graphs (*n* = 5) are shown on the left panel. PSC-amplitudes were normalized to control values (average of 20 PSCs preceding drug application). In control, absolute amplitudes of inward and outward currents (mean ± S.D) in this series of experiments were: -196.3 ± 92.6 pA; 193.1 ± 119.6 pA

On the other hand, in the solution with higher HEPES concentration (10 mM) the effect of **5b** (1 μM) on inward current amplitude was substantially smaller and not statistically significant (Fig. 4). On average the amplitude of inward PSCS in **5b** presence was 93.1 ± 5.3 % of control (*P* >0.25; T = -1.3; df = 4; paired Student’s t-test). Taken together, the above results suggest involvement of protons in the effect of **5b**.

This point can be also supported by our observation that currents that evoked *exogenous* GABA applications are not decreased in presence of **5b** (Additional file 1: Figure S2).

Nevertheless, to further confirm that the effect of **5b** is specific (related to its action on ASICs) we tested the effects of other chemically distinct ASIC blockers on PSCs at GABAergic synapses. For this purpose we used amiloride and diminazene [10, 24].

### Effects of amiloride and diminazene on GABAergic PSCs are similar to the effect of 5b

**Fig. 5:**
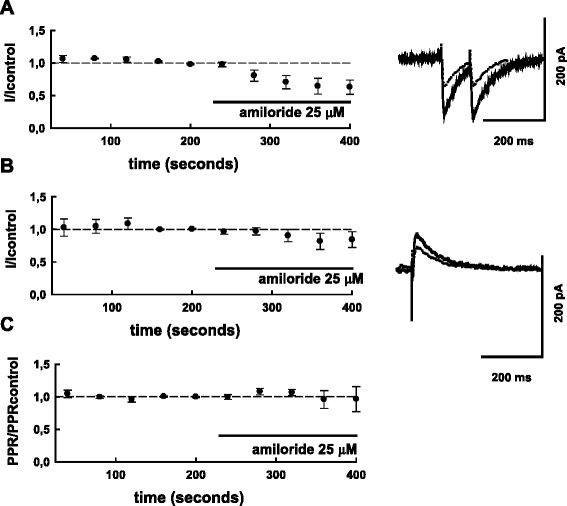
A non-selective blocker of ASICs amiloride suppresses inward GABAergic currents. In the same series of experiments during each sweep evoked PSCs (2 PSCSs with 100 ms interval) were recorded below their reversal potential as inward currents (**a**), and above the reversal potential, as outward ones (**b**), see Methods for details. Superimposed traces of original current traces (averages of 10 sequential PSCs) before (solid lines) and after (dotted lines) amiloride (25 μM) application are shown on the right panel, summary graphs (*n* = 5) are shown on the left panel. PSC-amplitudes and paired-pulse ratios for inward PSCs (PSC2/PSC1) were normalized to control values (average for 20 PSCs preceding drug application). Normalized paired-pulse ratio is plotted in (**c**). The experiments were done in ‘HEPES 2’ solution. In control, absolute amplitudes of inward and outward currents (mean ± S.D) in this series of experiments were: -174.4 ± 75.0 pA; 110.6 ± 60.8 pA

The experiments were performed in HEPES-2 solution. Experimental protocol was similar to that used to study the effect of **5b**, but during each sweep the presynaptic neuron was stimulated three times – two currents were recorded as inward and one as outward (see Fig. 1b and Methods for details). To examine possible involvement of presynaptic mechanisms in the effects [15, 27] the paired-pulse ratio (PPR) protocol was applied in these experiments. We found that following amiloride (25 μM) application, the inward currents were substantially decreased in comparison to the control (Fig. 5a). On average the inward current decreased to 69.5 ± 10 % (*n* = 5), and the decrease was statistically significant (*P* < 0.05; T = -3; df = 4; paired Student’s t-test). At the same time smaller decrease of outward currents was observed (Fig. 5b): on average the amplitude of outward PSCS in the presence of amiloride was 88.3 ± 9 % of control (*P* =0.6; T = -1.29; df = 4; paired Student’s t-test). Virtually no *shift* of estimated PSCs reversal potential (-1.33 ± 0.28 mV, *n* = 5) was observed in the presence of amiloride.

Equally no changes were observed in the paired-pulse ratio (IPSC2/IPSC1 of inward currents) which remained at 101,2 ± 7.5 % in the presence of amiloride.

**Fig. 6:**
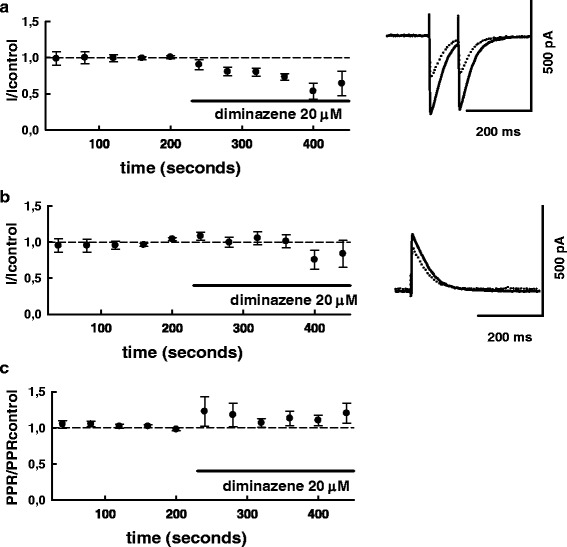
ASIC blocker diminazen suppresses inward GABAergic currents. In the same series of experiments during each sweep evoked PSCs (2 PSCSs with 100 ms interval) were recorded below their reversal potential as inward currents (**a**), and above the reversal potential, as outward ones (**b**), see Methods for details. Superimposed traces of original current traces (averages of 10 sequential PSCs) before (solid lines) and after (dotted lines) diminazene (20 μM) application are shown on the right panel, summary graphs (*n* = 6) are shown on the left panel. PSC-amplitudes and paired-pulse ratios for inward PSCs (PSC2/PSC1) were normalized to control values (average for 20 PSCs preceding drug application). Normalized paired-pulse ratio is plotted in (**c**). The experiments were done in ‘HEPES 2’ solution. In control, absolute amplitudes of inward and outward currents (mean ± S.D) in this series of experiments were: -188.5 ± 97.0 pA; 178.4 ± 58.4 pA

Similar to the effect of **5b** and amiloride, diminazene (20 μM) also inhibited the inward currents, while weaker effect on the outward currents was observed (Fig. 6a, b).

On average, the inward currents were decreased to 71.8 ± 4.2 % (*n* = 6) of the control, the decrease was statistically significant (*P* < 0.05; T = -6,77; df = 5; paired Student’s t-test). At the same time smaller decrease of outward currents was observed (Fig. 6b): on average the amplitude of outward PSCS in the presence of diminazene was 95,2 ± 7.2 % of control. The decrease was not statistically significant (*P* =0.54; T = -0.65; df = 5; paired Student’s t-test). No apparent *shift* of estimated PSCs reversal potential was observed in the presence of diminazene. (-1.76 ± 0.28 mV, *n* = 6).

The presence of diminazene did produce a weak enhancement of paired-pulse ratio (Fig. 6c) to 112 ± 9.25 % of the control. The difference was not statistically significant (*P* =0.25; T = -1.29; df = 5; paired Student’s t-test) but in view of the tendency towards the increase of paired-pulse ratio in the presence of diminazene, we have carried out additional (*n* = 8) experiments to check if the paired-pulse ratio is also changed in the presence of **5b** (1 μM). On average we observed a small enhancement of paired-pulse ratio to 106 ± 2.5 % of control value which was not statistically significant (*P* =0.25; T = 1.22; df = 7; paired Student’s t-test), (not illustrated). Thus, we did not reveal systematic changes of PPR in the presence of tested ASIC blockers. Since changes of PPR are thought to reflect involvement of a presynaptic mechanism in modulation of synaptic transmission [15, 27], these results argue against (though do not totally exclude) involvement of such a mechanism in our experimental conditions.

## Discussion

In spite of the fact that the first evidence indicating the presence of receptor for protons in the nerve cell membrane was obtained a long time ago [28] and tremendous progress in this field has been demonstrated in the subsequent studies [5, 9, 16, 29–32] the physiological role of ASICs is still far from being clear. Their involvement in regulation and plasticity of glutamatergic synaptic transmission in the hippocampus has been demonstrated. Protons are considered to be neurotransmitters regulating synaptic plasticity in the lateral amygdala [5]. In spite of growing evidence indicating that the density of ASICs is substantially higher in GABAergic interneurons than in glutamatergic cells [17] and recent demonstration of functional crosstalk between ASICs and GABA_A_-receptors [10, 11], the possible involvement of ASICs in the regulation of GABAergic transmission remained unclear. In our work we present evidence for the first time that ASICs play a functional role at hippocampal GABAergic synapses. This role is mediated, at least partially, by a postsynaptic but (predominantly) modulatory mechanism.

### Effects of ASIC blockers on GABAergic PSCs are due to their specific action

We found that GABAergic postsynaptic currents, recorded below their reversal potential as inward currents, are suppressed by all the employed blockers of ASICs. In the same cells the suppression of postsynaptic currents, recorded above their reversal potential as outward currents was statistically insignificant.

A possible explanation of the differential effect of ASICs antagonists on inward and outward GABAergic PSCs could be related to their *direct* voltage-dependent action on GABA receptors/channels. However this is unlikely because:

1. the antagonists are chemically different;
2. the effect of **5b** is attenuated in HEPES 10 solution, suggesting involvement of protons in the effect;
3. diminazene, amiloride and **5b** do not have any effect on the currents evoked by exogenous GABA application (again suggesting involvement of protons in the effect).

Apart from the chemical dissimilarity of amiloride and diminazene, they are structurally different [24], and mechanisms of their action on ASICs are different as well [24]. Similarly, amiloride and **5b** have different mechanisms of action on ASICs [21]. Indeed, **5b** is an orthosteric antagonist of ASIC1 [21] while amiloride is an open channel blocker [33].

Observed in our experiments attenuation of the **5b** effect in HEPES 10 solution, suggests involvement of protons in the effect. This suggestion is in line with the lack of effects of diminazene, amiloride and **5b** on currents evoked by exogenous GABA. Indeed, according to previous observations amiloride (100 μM) and diminazene (50 μM) do not affect (inward) currents induced by exogenous GABA applications (GABA-responses) [10]. This is also true for **5b:** inward GABA-responses were not affected in presence of 1 μM **5b** (Additional file 1: Figure S2).

Additionally, we would like to mention that although amioloride is known to be not selective at high concentrations (for instance [34]), at concentration (25 μM) used in our experiments amiloride was shown to be a potent antagonist, mainly for ASIC receptors. As far as we know, diminazene is rather selective against ASICs within the time scale of our experiments (minutes). It does target DNA [35], but related consequences of this action shouldn’t be expected within minutes. Indeed, we are not aware of any other than ASICs targets of diminazene, which could be responsible for ‘rapid’ side effects.

Despite an extensive search for other than ASIC targets of **5b**, this compound at 1 μM concentration was found only to affect (slightly) NMDA currents (see supporting information for [21]), which should not be a concern for our experiments because they were performed in the presence of an NMDA receptor blocker (APV) and the NMDA receptor was accordingly already blocked.

Additionally, our results regarding magnitude of effects of **5b** (1 μM), amiloride (25 μM) and diminazene (20 μM) on PSCs are in reasonable agreement with expected effects of these blockers on ASIC currents in hippocampal neurons (please see Additional file 1: Figure S3 for the expected effects).

Finally, specificity of the effects of ASIC blockers on GABAergic PSCs is in concert with previously reported results obtained using ASIC1 knockout animals [6]. Indeed, about 20 % decrease of inward GABAergic PSCs was observed in hippocampal neurons from ASIC1 knockout as compared to unmodified animals. While this change was not found to be statistically significant (*P* = 0.27) [6], the lack of significance may reflect larger variability and lower power of unpaired statistical tests together with small magnitude differences.

Taken together, all these results strongly argue against direct *unspecific* effect of the three tested ASICs antagonists on GABA receptors/channels and suggest involvement of protons in the effects of the ASICS antagonists on GABAergic PSCs.

### The suppressing effect of the blockers of acid-sensing ion channels on GABAergic transmission is due, at least partially, to a postsynaptic but (predominantly) modulatory mechanism

It may be reasonably assumed, that if synaptic transmission is affected by a chemical via a purely presynaptic mechanism, similar changes of postsynaptic currents recorded below and above PSC reversal potential, would be expected. We found, however, that while GABAergic PSCs, recorded below their reversal potential as inward currents, are suppressed by all the employed ASIC blockers, in the same cells the suppression of outward currents was statistically insignificant. These results imply that the effects of blockers in our experiments are at least partially postsynaptic. On the other hand, direct involvement of ASICs in PSCs generation documented for lateral amygdala neurons [5], does not seemingly occur in hippocampal neurons [6, 36]. Our results tend to agree with the latter observations. Indeed, under our experimental conditions, if a substantial fraction of synaptic current is mediated by ASICs, a decrease in the net inward current, and an *increase* in the net outward current would be expected once ASICs are blocked. While in the presence of ASIC antagonists we did observe a decrease of the inward currents and a small *decrease* in the outward currents. Lack of substantial direct involvement of ASICs in PSCs generation in our experiments can be also supported by comparing the possible relative contribution of ASIC current to total synaptic current. Based on the results of our experiments with application of bicuculline alone, the relative contribution of ASIC currents to total synaptic current is less than 9 % (8.29 ± 2.24). Our experiments using 5b and suramin suggest an even lower percentage. Indeed, the residual currents were nearly unaffected in presence of 5b (1 μM) but strongly suppressed (to 16.9 ± 4.3 % of control) by suramin at 200 μM concentration. Since suramin (500 μM) does not affect ASIC currents in hippocampal neurons [24] the above results *also* indicate that relative contribution of ‘synaptic’ ASIC current is much smaller than 9 %. At the same time, the relative magnitude of the effects produced by ASIC blockers on synaptic currents recorded in the absence of bicuculline is about 20-30 %. These results, taken together, strongly support our point that the suppressing effect of the ASICs blockers on GABAergic transmission is due, *predominantly* to a modulatory mechanism. Additionally, we would like to mention that absolute amplitude of proton-mediated synaptic currents in amygdala is about 7-10 pA [5]. Since the density of proton-activated currents (evoked by pH shift to 5) is ~ 75 pA/pF in amygdala and 20 pA/pF in hippocampus [8], a much reduced proton-mediated component of synaptic current in the hippocampus may be expected. In our experiments, however, the absolute value of inward synaptic current suppressed by ASIC antagonists is about 40—60 pA. As for the current *mediated* by ASICs in hippocampal GABAergic synapses, we believe this was undetectable due to its small absolute and relative amplitude.

### Potential mechanism of the modulatory effect

Functional interaction between ASICs and GABA_A_-receptors in isolated neurons has been recently demonstrated [10, 11]. Activation of GABA_A_-receptors strongly changed ASIC-currents amplitude and pharmacological sensitivity [10], and the effect was blocked by antagonists of GABA_A_ receptors [10]. On the other hand, a modulatory effect of ASIC activation on GABA_A_-currents was also observed in HEK293 cells co-transfected with GABA_A_ and ASIC1a or in primary cultured DRG neurons. The immunoassays showed that both GABA_A_ and ASIC1a proteins were co-immunoprecipitated mutually either in HEK293 cells co-transfected with GABA_A_ and ASIC1a or in primary cultured DRG neurons [11]. These data suggest direct protein-protein mechanism of interaction between GABA_A_ and ASICs. This suggestion is also indirectly supported by the observation that modulatory effect of GABA_A_-receptors activation on ASICs-currents can be observed in excised patches [10]. We assume that an interaction between ASICs and GABA_A_-receptors is quite likely to occur at GABAergic synapses upon acidification at the synaptic cleft. This assumption can be supported by the *lack* of the apparent effect of **5b** on inward PSCS in the *presence* of bicuculline, observed in our experiments. Indeed, this should be expected if the effect of **5b** on inward PSCS in the *absence* of bicuculline is due to crosstalk between ASICs and GABA_A_-receptors, because the crosstalk in isolated neurons was blocked by antagonists of GABA_A_-receptors- receptors bicuculline and picrotoxin [10].

Within the framework of this assumption, differential effects of the ASICs antagonists on inward and outward PSCs which we observed in our experiments would indicate that this interaction is voltage-dependent. In this regard, possibility to alter GABA-currents decay by changing voltage [37, 38] and activation of ASICs [11] may be not just a coincidence. It is worth noting that feature of voltage-dependence of interaction between receptors is, of itself, not very surprising. Indeed, a physical link between group-I metabotropic glutamate receptors and NMDA receptors results in a functional crosstalk, which is voltage-dependent [39]. Nevertheless we cannot currently exclude a possibility that the modulation of PSCs by ASICs under our experimental conditions depends on the direction of the current, rather than being intrinsically voltage-dependent. This, however, is less likely because in isolated neurons the interaction of ASICs and GABAA receptors does not depend on the direction of the GABA-current [10].

Thus, we currently suggest that the effects we observed in our experiments are due to functional crosstalk between ASICs and GABA_A_-receptors reported recently in isolated neurons [10, 11]. Nevertheless, further verification of this suggestion is necessary.

#### To summarize explanation of our results

Both, GABA and protons are released by presynaptic GABAergic neurons upon stimulation and diffuse to the postsynaptic membrane. The protons, which are smaller, arrive to the postsynaptic membrane first and: (i) activate ASICS; (ii) modulate GABAA receptors.

The modulatory effect of protons is caused by an interaction of GABAA receptors and ASICs similar to that described in previously published papers.

Both, synaptic currents directly mediated by ASICs and the modulation of GABAA receptors are suppressed by ASIC blockers. We, however, resolve predominantly modulatory effect, as more potent.

We hypothesize that interaction of GABAA and ASICs is voltage-dependent because statistically significant effects of blockers were observed only at more hyperpolarized potentials, at which currents are recorded as inward.

##### To conclude

ASICs are abundant in many brain areas and are known to have important physiological functions. However, because of rapid desensitization of ASIC-mediated currents, synapses are among the few places where they can be activated under physiological conditions and, thus, mediate their physiological roles. In this work we demonstrated for the first time that three structurally different ASIC blockers affect GABAergic PSCS in a similar manner, strongly suggesting that ASICs are involved in regulation of GABAegic synaptic transmission under physiological conditions. Considering our results and previously published data, we conclude that the effect of the ASIC blockers on GABAegic synaptic transmission is due to an at least partially postsynaptic but (predominantly) modulatory mechanism. Our results may be of importance for applied pharmacology, because ASICs are considered as therapeutic targets for neurological diseases and ASIC blockers as potential neuroprotectors [40].

## Additional file

Additional file 1: Supplemental **Figures S1**-**S3**. Additional info about compound 5b. (DOC 1812 kb)

## Abbreviations

5b (compound): 2-oxo-2H-chromene-3-carboxamidine derivative
APV: DL-2-amino-5-phosphonovaleric acid
ASICs: Acid-sensing ion channels
CNQX: 6-cyano-7-nitroquinoxaline-2,3-dione-
GABA: Gamma -aminobutyric acid
HEPES: N[2-hydroxyethyl]piperazine-N’-[2-ethanesulfonic acid]
PSCs: Postsynaptic currents
