# Supplementary material for: A modulatory role of ASICs on GABAergic synapses in rat hippocampal cell cultures
Source: Mol Brain. 2016 Oct 19;9:90. doi: 10.1186/s13041-016-0269-4 (PMC5070181; doi:10.1186/s13041-016-0269-4)
Supplement: Additional file 1: — Supplemental Figures S1-S3. Additional info about compound 5b. (DOC 1812 kb) [file 13041_2016_269_MOESM1_ESM.doc]

# Supplementary Material

**Additional details regarding 5b**

(Dr. O. Maximyuk personal communication).

‘We have tested the ability of compound 5b to inhibit rat ASIC1a-like, ASIC3-like and ASIC2-like currents in acutely isolated. We found that 10 µM of 5b produce virtually the same inhibition on native fast ASIC1a-like (48.3±5.0 % [n=4], hippocampal neurons of rat) and native fast ASIC3-like (45.2±4.3 % [n=4], DRG neurons of rat) when these currents were elicited by pH drop from 7.4 to 5.0. Unlike fast and highly proton sensitive ASIC1a and ASIC3 currents, slow ASIC2 currents were insensitive to 10 µM of 5b (99.3.2±1.3 %, n=3). The native currents can be mediated in part by heteromers of ASIC1a and ASIC3 with ASIC2.’

**Figure S1**. **Suramin suppresses bicuculline-resistant (residual) currents at GABAergic connections.**

**A.** Superimposed traces of original current traces (averages of 10 sequential PSCs) before and after suramin (200 μM) application. B**.** Summary graph (n=4)

In control (before suramin application), absolute amplitude of currents (mean ± S.D) in this series of experiments was: -108.7±62.7 pA.

5b does not decrease currents, induced by exogenous GABA applications in hippocampal neurons.

To examine possible direct influence of **5b** on GABA receptors we studied effect of this compound on currents, induced by exogenous GABA application. Methods used for these experiments are described in a previous study [1]. Briefly, GABA-activated currents were elicited by rapid application of GABA (100 µM) delivered from a multi-barrel fast perfusion system for 3 s every 2 minutes. **5b** was pre-applied with the external solution containing no GABA for 1-2 min and then applied together with GABA. Examples of original traces, recorded before **5b** (1 μM) application and in its presence are shown in Fig. S2 A; a graph summarizing data is shown in Fig. S2 B. On average, amplitude of GABA-currents in the presence **5b** was 102.6 ±2.25% of control (P=0,29; paired t-test n=6).

Figure S2. 5b does not decrease currents, induced by exogenous GABA applications in hippocampal neurons.

A) Examples of original traces, recorded before **5b** (1 μM) application and in its presence.

B) Summary graph of 5b effect on GABA-induced currents (n=6).

GABA-induced currents in control and in its presence.

In control (before **5b** application), absolute amplitude of currents (mean ± S.D) in this series of experiments was: -1012.4±751.2 pA

**Figure S3. Estimated* effects of 5b (1 µM), amiloride (25 µM) and diminazene (20 µM) on ASIC- currents, evoked by mild (to pH 6,7) acidification.**

* The effect of **5b** (1 µM) was actually measured in amygdala neurons. It was found that ASIC-currents, evoked by mild acidification are decreased by 37.5±4.6% (P<0.01; T=-8,1; df=8); paired Student’s t-test; Kondratskaya, unpublished observations). Although density of ASIC currents in amygdala neurons is substantially higher than that in hippocampal cells [2], in terms of relative proportion of functional ASIC1a homomers, 1a:2a heteromers and 2a homomers cells from both structures are expected to differ only slightly [3]. It is worth mentioning that the suppressing effect of **5b** (1 µM) on *total* ASIC currents, evoked by mild (to pH 6,7) acidification (37,5%), is substantially weaker than those on *ASIC1A* and native *ASIC1A-lke currents* (~90%) [4]. For ASIC currents, evoked *by mild* (to pH 6,7) acidification the mismatch is rather surprising, because these currents should be predominantly mediated by ASIC1A homomers.

* The effects of amiloride and diminazene were estimated based on the following considerations**.**

The IC50 of **amiloride** on ASIC current is reported to be 10–20 μM [5,6]. Thus, the currents should be decreased at least by 50% by amiloride at 25 µM concentration (approximately, by 60-70%).

**Diminazene** (3 µM) decreased ASIC1a current by 39 %, ASIC1b current by 92 %, ASIC2a current by 51 % (*n* = 8, *p* < 0.01), and ASIC3 current by 74 %. [7]. Thus, nearly complete block of ASIC-currents should be expected in the presence of diminazene at 20 µM concentration. Indeed, diminazene (20 µM) blocked ASIC-currents by 97% [8].

This comparison suggests that effects of **5b** (1 µM) and amiloride (25 µM) on *PSCs* in our experiments should be about 37% and 65 % as compared to the effect of diminazene (20 µM). The *observed* effects were ~ 60% for **5b** (1 µM) and ~100% for amiloride (25 µM). Although the match is far from being perfect, it still reasonably explains the weaker effect of **5b** (1 µM) as compared to those of amiloride (25 µM) and diminazene (20 µM). The quantitative differences are also explainable. For instance, considering that effect of **5b** on ASIC-current strongly depends on pH [4], larger effect would be expected for milder pH drops (e.g. at pH 6,8), which are also relevant for synaptic cleft acidification [9]. Although amiloride is a non-competitive blocker of ASICs, it cannot be excluded that it is more potent at pH 6,7 (the IC50 values of amiloride on ASIC current we used in our estimate were measured at pH 5).

Reference List

1. Kondratskaya E, Shin MC, Akaike N: Neuronal glutamate transporters regulate synaptic transmission in single synapses on CA1 hippocampal neurons**.** *Brain Res Bull* 2010, 81**:** 53-60.

2. Wemmie JA, Askwith CC, Lamani E, Cassell MD, Freeman JH, Jr., Welsh MJ: Acid-sensing ion channel 1 is localized in brain regions with high synaptic density and contributes to fear conditioning**.** *J Neurosci* 2003, 23**:** 5496-5502.

3. Zha XM: Acid-sensing ion channels: trafficking and synaptic function**.** *Mol Brain* 2013, 6**:** 1.

4. Buta A, Maximyuk O, Kovalskyy D, Sukach V, Vovk M, Ievglevskyi O *et al*.: Novel Potent Orthosteric Antagonist of ASIC1a Prevents NMDAR-Dependent LTP Induction**.** *J Med Chem* 2015, 58**:** 4449-4461.

5. Wu LJ, Duan B, Mei YD, Gao J, Chen JG, Zhuo M *et al*.: Characterization of acid-sensing ion channels in dorsal horn neurons of rat spinal cord**.** *J Biol Chem* 2004, 279**:** 43716-43724.

6. Waldmann R, Champigny G, Bassilana F, Heurteaux C, Lazdunski M: A proton-gated cation channel involved in acid-sensing**.** *Nature* 1997, 386**:** 173-177.

7. Chen X, Qiu L, Li M, Durrnagel S, Orser BA, Xiong ZG *et al*.: Diarylamidines: high potency inhibitors of acid-sensing ion channels**.** *Neuropharmacology* 2010, 58**:** 1045-1053.

8. Chen X, Whissell P, Orser BA, MacDonald JF: Functional modifications of acid-sensing ion channels by ligand-gated chloride channels**.** *PLoS One* 2011, 6**:** e21970.

9. Palmer MJ, Hull C, Vigh J, von GH: Synaptic cleft acidification and modulation of short-term depression by exocytosed protons in retinal bipolar cells**.** *J Neurosci* 2003, 23**:** 11332-11341.
